# Novel Management of a Femoral Fracture in Klippel-Trenaunay Syndrome

**DOI:** 10.7759/cureus.26652

**Published:** 2022-07-07

**Authors:** Nandesh C Patel, Shakir Hussain, Usman Fuad, Edward Spurrier

**Affiliations:** 1 Trauma and Orthopaedics, Queen Elizabeth Hospital Birmingham, Birmingham, GBR

**Keywords:** case report, celox, flexible intramedullary nail, fracture, klippel-trenaunay syndrome

## Abstract

Klippel-Trenaunay syndrome (KTS) is a rare congenital disorder with a clinical triad of capillary malformations, vascular abnormalities, and bone/soft tissue hypertrophy. This is the first case of closed femoral shaft fracture in a patient with KTS managed by flexible intramedullary nails.

A 34-year-old patient sustained a right femoral mid-shaft spiral fracture after slipping on the grass. Due to a very narrow femur and large venous malformations, nail or plate fixation was impossible. Surgery was conducted using flexible intramedullary (TENS) nails with good reduction but significant bleeding which was controlled with tranexamic acid and CELOX. The patient required 4 units of red blood cells, 3 units of fresh frozen plasma, and 900 mL of cell saver intraoperatively with a further 2 units of RBC post-op. Fracture union was achieved 14 months after the initial fracture with additional pulsed ultrasound therapy.

Bleeding from vascular malformations during surgery makes operative management challenging in KTS patients. Previous studies have reported a variety of management strategies to achieve fracture fixation and union including IM nailing, plate fixation, and external fixators, but encountered significant bleeding of up to 10 units and 15 units, respectively. Ultrasound therapy has been utilized as a useful adjunct in lower limb fracture with delayed therapy.

Management of fractures in patients affected by KTS is extremely challenging despite extensive workup and planning to evaluate the optimal fixation method and explore strategies to reduce the risk of intra-operative bleeding. Management strategies should be tailored to the patient with close follow-up to assess fracture union.

## Introduction

Klippel-Trenaunay syndrome (KTS) is a rare congenital disorder of the vascular system characterized by the clinical triad of capillary malformations, vascular abnormalities, and bone and/or soft tissue hypertrophy [1]. It mostly involves the lower limb but has been reported to involve the upper limb and trunk [2]. Arterio-venous fistulas are rarely found in the affected limb, and if present the syndrome is then called Klippel-Trenaunay-Weber syndrome [3].

A fracture in the affected limb in patients with KTS can be difficult to manage; bones are abnormal, osteoporotic, and often narrow leading to a significant risk of profuse bleeding. There are only a few case reports in the literature on the management of fractures in this rare condition [3-6] with no consensus on management techniques [3,4].

We are reporting the first case of a closed femoral shaft fracture in a patient with KTS managed with a flexible nail including strategies to reduce intra-operative bleeding.

## Case presentation

This 34-year-old Caucasian gentleman, diagnosed with KTS at birth, presented to the emergency department (ED) in November 2019 with right thigh pain and deformity after slipping on the grass in his garden. The patient has a long orthopaedic history with pain whilst weight-bearing on his right affected leg and has been using crutches to mobilise since the age of 10. The patient requires orthotic shoes for a clinically shorter limb from a right knee contracture. He had corrective surgery with an Ilizarov frame at 16, requiring multiple transfusions for pin site bleeding, which partially improved a flexion deformity in the knee. He mobilises partially weight-bearing on this leg with intermittent pain requiring regular analgesia. The patient was in employment and independent for activities of daily living. The patient had no other significant past medical history or complications of KTS other than occasional bleeding from scabs on his scrotum and feet. At presentation, the patient had right thigh pain with no open wounds and was neurovascularly intact.

Investigations

**Figure 1:**
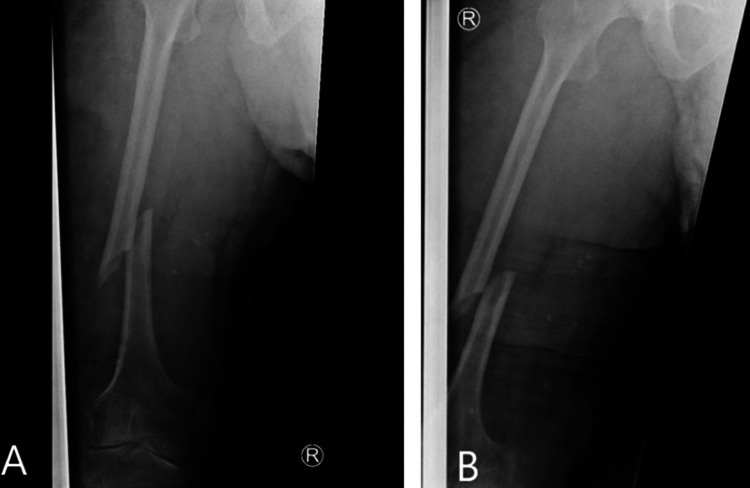
Radiographs showing the initial fracture pattern with anteroposterior (A) and lateral (B) views

An x-ray in ED (Figures 1A, 1B) showed a displaced spiral fracture of the right femoral shaft. Skin traction was applied, and the patient was admitted under orthopaedics initially for retrograde intramedullary nailing using a paediatric kit. Previous historical MRI images showed extensive vascular malformations in the affected limb, pelvis, and scrotum, involving all layers including skin, subcutaneous tissue, and the muscular compartment.

Treatment

Pre-operative Planning

**Figure 2:**
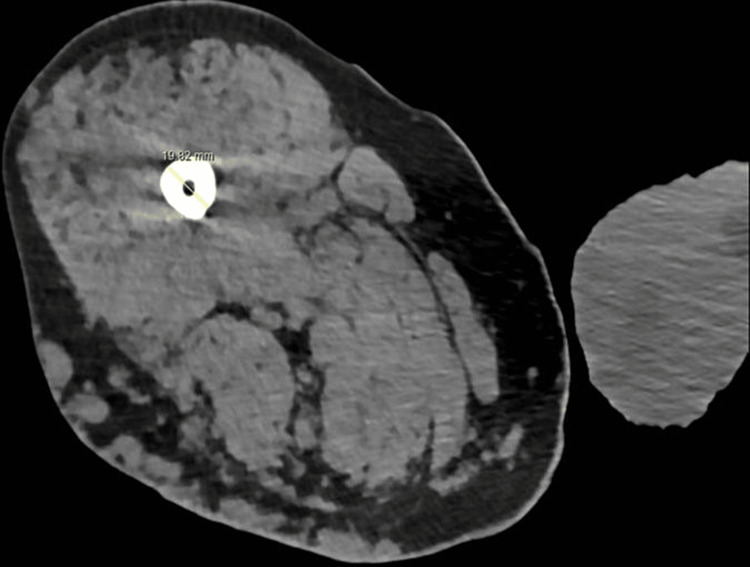
CT scan showing narrow femoral canal

A CT scan of the femur was done to assess the fracture pattern and the medullary canal diameter, which was 4.6 mm at its narrowest point (Figure 2). An ultrasound of the leg was used to identify an operative window and showed large varicosities in the posterior and lateral thigh. The aim of treatment was to restore the patient to pre-fall mobility and function. The patient was discussed with paediatric orthopaedic, vascular and haematology colleagues. It was decided that the patient was best treated with flexible intramedullary nailing due to the narrow medullary canal with a plate revision should this fail. Pre-operative clotting was normal and haematology colleagues ensured that massive perioperative transfusion would be available if required.

Procedure

**Figure 3:**
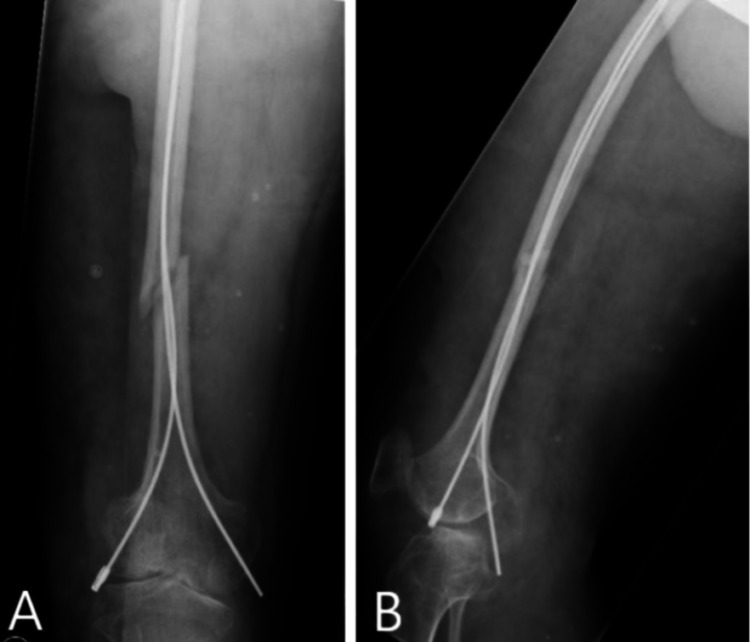
Radiographs immediately after reduction of fracture showing anteroposterior (A) and lateral (B) views

The patient was operated on with a vascular surgeon present during the procedure. The patient was laid supine on the table. Small medial and lateral longitudinal incisions were made as no safe planes were able to be identified in any of the tissue layers. The fascia was cautiously dissected using diathermy and ligasure, despite this there was persistent bleeding. The patient was given 1g of tranexamic acid and the wound was packed with Celox gauze with pressure applied to control the significant bleed. Medial and lateral bone entry was made, and 2.5 mm flexible intramedullary nails were passed under II using an F reduction tool to the proximal femur with good fracture reduction achieved (Figures 3A, 3B). We used TENS (Titanium Elastic Nail System, DePuy Synthes, J&J, USA) and the nail was rotated to maximise elastic reduction. The patient was closed using fibrillar haemostat, vicryl and monocryl and a wool and crepe bandage. The patient required 4 units of red blood cells (RBC), 3 units of fresh frozen plasma (FFP) and 900 mL of autologous blood transfusion from a cell saver intraoperatively.

Post-operative Recovery

The patient remained stable after surgery and started post-operative rehabilitation. The knee was immobilized in a brace for two weeks and kept non-weight bearing, with the view to change to a hinged brace after two weeks upon x-ray. The aim was to discharge the patient once mobile. The patient’s haemoglobin dropped from 156 g/L to 104 g/L immediately post-operatively although he remained asymptomatic. The patient required 2 units of RBC on post-op day five as symptomatic anaemia was limiting the patient’s mobility. The patient developed total right-sided sensorineural hearing loss on day four. The patient was discharged on day 13 with community physiotherapy, ENT, and orthopaedic follow-up in the community.

Outcome and follow-up

**Figure 4:**
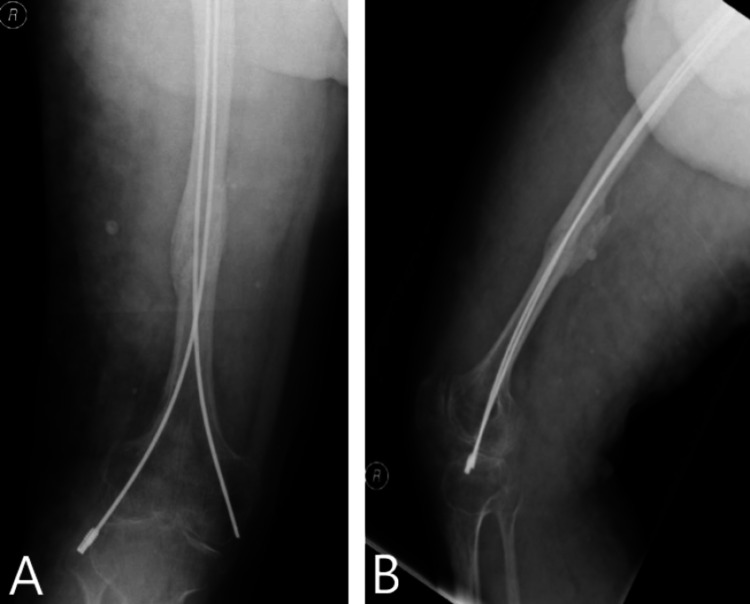
Radiographs showing radiological union of fracture with anteroposterior (A) and lateral (B) views

The patient was reviewed in a clinic in January 2020, which showed delayed wound healing and posterior callus formation. The patient’s physiotherapy, which focused on knee mobility exercises, was limited by pain. The patient was started on EXOGEN (Bioventus, USA) low-intensity pulsed ultrasound therapy due to delayed fracture healing in March 2020. The medial nail was shortened and buried in June 2020 under GA with no immediate complications. In February 2021, the patient achieved clinical and radiological union (Figures 4A, 4B).

## Discussion

We present a case of a 34-year-old patient with KTS who sustained a complete spiral fracture of the right femur, stabilised using flexible intramedullary nailing. This is the first reported case of a femoral fracture in an adult KTS patient, fixed with paediatric, flexible intramedullary nails. KTS is not usually life-threatening, and management of the condition is conservative with lifelong follow-up [7]. However, vascular and bony malformations may lead to challenges in managing patients requiring orthopaedic surgery [4].

Multiple options were considered when deciding the optimal fracture fixation technique for this patient. Previous literature has reported the use of adult-sized intramedullary nailing [5,6], external fixation [3], and plate fixation [4] in KTS patients with femoral fractures. Conservative management has also been attempted with poor results [6]. Adult-size titanium Intramedullary nailing was considered inappropriate due to the patient’s narrow femoral canal. Plating had the highest risk of intra-operative bleeding due to the extent of the wounds. Similarly, external fixation was considered but deemed unsafe due to the patient’s fragile skin and risk of bleeding, given the patient’s history of bleeding profusely from pin sites. Conservative management was not possible due to the substantial risk of non-union due to the patient’s dysplastic femur and fracture pattern. As a result, the flexible intramedullary nail was the safest option, with plate fixation if nailing failed. Accounting for the limitation in its mechanical strength when compared to intramedullary nails, we used pre-bending of the nails in our procedure, as this has been shown to increase fracture stability [8].

Bleeding from vascular malformations in KTS is a well-reported challenge in the operative management of fractures. Tsaridis et al. reported the first case of a femoral shaft fracture in a 42-year-old female with KTS fixed with an IM nail and required 10 units of RBCs during and after surgery [5]. Notarnicola et al. reported management in a 52-year-old male with an IM nail only requiring 4 units of RBCs [6]. Union was achieved in both these cases. Nahas et al. presented a similar case in a 32-year-old male initially treated with low-intensity pulsed ultrasound but subsequently requiring plate fixation due to non-union. This patient required 15 units of RBCs during surgery [4]. Gupta et al. used external fixator as definitive management and this patient had complete union of the fracture [3]. Our patient required 6 units of RBC during his admission, including 900 mL of autologous blood transfusion, despite tranexamic acid, careful planning, and dissection during the surgery. Although difficult to compare directly, using flexible intramedullary nailing seems to have lowered our transfusion requirement compared to other reported cases.

Similar to the case reported by Nahas et al. [4], our patient also used ultrasound therapy although required a significantly longer time on it. Pulsed ultrasound therapy has been shown to be useful in the healing of lower limb fractures with delayed union after surgery [9]. This adds to the evidence that pulsed ultrasound can be useful in fracture healing for patients with KTS.

Our patient suffered from permanent sudden-onset sensorineural hearing loss following his surgery. This is a rare phenomenon seldom reported post-non-otological surgery. Many of these cases occur in cardiopulmonary bypass surgery, making it even rarer following orthopaedic surgery. The most likely aetiology for unilateral hearing loss in non-otological surgery is microemboli, such as particulate matter, or fat, causing ischaemia of hair cells within the ear [10].

## Conclusions

In conclusion, femoral fracture management in patients with KTS is complex and requires extensive pre-operative planning. Timing of surgery and implant choice is vital to maximise chances of fracture union. Extensive bleeding during surgery is a recognised risk with no established techniques to reduce this, nevertheless, efforts should be made to minimise bleeding. Management strategies should be tailored according to fracture pattern, femoral canal diameter and current skin and vasculature within the affected limb. Close follow-up is essential to assess fracture union due to the high non-union risk in these patients.
